# Scientific Analysis of the Polychrome Paintings on the Stele Pavilion of Guandi Temple in Shanxi, China

**DOI:** 10.3390/ma19173672

**Published:** 2026-08-29

**Authors:** Luxi Li, Xilin Wang, Shengjie Wu, Fangzhou Liu, Ran Liang, Ming Tang

**Affiliations:** 1 National Museum of China, Beijing 100006, China; lee1572026@163.com (L.L.); wxl5702026@163.com (X.W.); 2 School of Cultural Heritage and Information Management, Shanghai University, Shanghai 200444, China; sj-wu18@tsinghua.org.cn; 3 Academy of Arts & Design, Tsinghua University, Beijing 100084, China; liufz21@tsinghua.org.cn (F.L.); liangr22@tsinghua.org.cn (R.L.)

**Keywords:** Guandi Temple, architectural polychrome, Qing dynasty, pigment analysis, ground layer materials

## Abstract

**Highlights:**

- The pigments used in the polychrome paintings include cinnabar, iron red, minium, lavendulan, ultramarine, indigo, and carbon black.
- The organic binders identified in the ground layers are tung oil, rosin, animal glue, and pig blood, with distinct formulations for oil and polychrome decorations.
- The oil decoration and polychrome paintings show repainting and ground-layer modifications, indicating at least two construction phases.
- Arsenic was detected in multiple pigment layers and in association with lavendulan, suggesting possible mobilization of soluble arsenate species within the painted structure.

**Abstract:**

The stele pavilion at the Jiezhou Guandi Temple in Shanxi Province is a significant Qing-dynasty architectural structure within one of China’s most renowned temple complexes dedicated to Guan Yu. However, its polychrome paintings have suffered severe deterioration due to prolonged weathering, and the composition, technological characteristics, and historical alterations of these paintings remain poorly understood. In this study, six polychrome pigment samples and one red oil-coating sample collected from different structural parts of the pavilion were systematically analyzed using laser Raman spectroscopy, scanning electron microscopy with energy-dispersive spectroscopy, X-ray diffraction, and pyrolysis-gas chromatography/mass spectrometry. The results show that the pigment layers primarily consist of mineral pigments including iron red, cinnabar, red lead, iron yellow, lavendulan, carbon black, and ultramarine, together with the organic pigment indigo. The oil coatings and polychrome paintings were restored in different periods. The initial oil coating, applied directly to the wood surface, has completely fallen off, whereas the later reapplication employed a single-layer ash ground with calcite aggregate and a binder of heat-bodied tung oil and rosin, indicating a simplified formula. The paint ground layer, dominated by kaolinite, contains pig blood, drying oil, and rosin, reflecting continuity with the Shanxi blood-based ground preparation tradition. These findings provide essential material evidence for conservation and offer a valuable case study for the technological history of Qing-dynasty regional architectural paintings in Shanxi.

## 1. Introduction

The protection of ancient architectural polychrome paintings has become an increasingly urgent concern in China, as prolonged outdoor exposure and environmental degradation continue to threaten these irreplaceable cultural assets. Among the diverse regional traditions of Chinese architectural decoration, the Qing-dynasty folk paintings of Shanxi Province remain comparatively understudied. This is despite their distinctive material choices and construction techniques, which differ markedly from the well-documented official styles centered on the Forbidden City.

The Jiezhou Guandi ancestral temple, originally established in 589 AD, is recognized as the earliest, largest, and best-preserved extant temple dedicated to Guan Yu in China. The stele pavilion, located on the eastern side of Chongning Hall within this temple complex, is a Qing-dynasty structure added to accommodate a poetry stele inscribed in 1734 by Prince Guoqin. Field investigations indicate that neither the pavilion nor its polychrome decorations have undergone documented restoration, and the oil-coated paintings have severely deteriorated, with only fragmentary remains surviving.

Qing-dynasty architectural polychrome paintings are broadly classified into official and folk categories according to their materials and workmanship. Official-style paintings were executed in strict compliance with the Engineering Methods and Imperial Engineering Methods promulgated in 1734, with specifications varying according to building rank. Consequently, research on official-style polychromes is relatively comprehensive. Numerous scientific studies have focused on the Forbidden City as the primary example [1,2,3,4], with additional investigations covering mausoleum architecture [5] and religious structures [6] that follow official protocols.

By contrast, folk architectural paintings in Shanxi Province typically employed locally sourced materials, using native yellow earth as the ground layer and adopting a single-layer ash application technique. However, scholarly attention to Jin-school architectural paintings remains limited. Existing studies on Shanxi’s vernacular polychromes have predominantly addressed aspects of architecture [7], art history [8], and historical documentation [9], while material and technological investigations have largely been confined to wall paintings [10,11,12] and painted sculptures [13]. Few studies have examined the materials and techniques of wooden architectural polychromes in Shanxi. Notable exceptions include the work of Guo Rui [14], who conducted scientific analyses of oil-coated paintings from Daoist, Buddhist, and residential buildings across various regions, and that of Dan Huang et al. [15], who detected tung oil as a primer in the Cangjie Temple wood paintings, along with cinnabar, lapis lazuli, lead white, emerald green, carbon black, and a proteinaceous adhesive. These isolated case studies, while valuable, have not yet coalesced into a systematic understanding of the material traditions, technological lineages, or regional variations characteristic of Shanxi folk architectural painting.

Scientific analysis has proven indispensable in the study of architectural polychrome materials and techniques. Researchers generally adopt integrated analytical approaches [16,17,18] to investigate microstructure, elemental composition, and molecular signatures, enabling not only pigment identification but also inferences regarding restoration chronology based on changes in material types.

Given the relatively abundant surviving polychrome remains on the stele pavilion, this study employed multiple analytical techniques, including three-dimensional video microscopy, scanning electron microscopy with energy-dispersive spectroscopy, Raman spectroscopy, X-ray diffraction, infrared spectroscopy, and pyrolysis-gas chromatography/mass spectrometry. A total of seven samples of oil-coated polychrome and ground layer materials from the pavilion were analyzed. This study aims to: (1) identify the pigment palette and binding media of the polychrome paintings, (2) distinguish between original construction and later restoration phases based on material differences, and (3) elucidate the technological lineage of Shanxi vernacular architectural painting.

## 2. Materials and Methods

### 2.1. Sample Collection

To obtain compositional information on the ground layer materials from different structural components, seven samples of oil-coated polychrome paintings were collected from various locations on the stele pavilion of the Guandi Temple in Yuncheng, Shanxi Province. The sampling information is summarized in Table 1. Photographs taken during sampling reveal that the oil-coated polychrome paintings on the stele pavilion have undergone severe deterioration, with only a few pigment-bearing areas remaining intact. The oil-coating samples are blocky solids, whereas the polychrome pigment samples are relatively loose, thin, and powder-like.

### 2.2. Experimental Methods and Instrumentation

#### 2.2.1. Micro-Morphology Examination

Samples GB-03 and GB-13 were embedded in epoxy resin and subsequently ground and polished using silicon carbide papers of progressive grit sizes and polishing cloths until a mirror-like surface was achieved. After preparation, the cross-sections were examined under a Keyence three-dimensional video microscope (KEYENCE, Osaka, Japan).

#### 2.2.2. SEM-EDX Analysis

Scanning electron microscopy and energy-dispersive spectroscopy (SEM-EDS) were performed using an XL benchtop scanning electron microscope (Phenom World B.V., Eindhoven, The Netherlands) equipped with a CeB_6_ filament, an accelerating voltage of 15 kV, and a backscattered electron detector operating in image mode. Microstructural observation and elemental composition analysis were conducted under high-vacuum mode, with an acquisition time of no less than 60 s for each energy-dispersive spectrum. Gold sputter-coating was applied only to the polished cross-section samples (GB-03 and GB-13), which were embedded in resin. The remaining pigment samples (GB-05, GB-07, GB-09, GB-15, and GB-17) were analyzed without gold coating, as they were collected as loose powders and mounted directly on conductive carbon tape.

#### 2.2.3. Raman Spectroscopy

Raman spectroscopy was performed on the pigment layers of six samples (GB-03, GB-07, GB-09, GB-13, GB-15, and GB-17), to identify the chromophoric mineral phases. Raman spectroscopic analysis was carried out using a LabRAM HR Evolution laser confocal Raman spectrometer (HORIBA, Palaiseau, France) equipped with an open sample stage. A 473 nm laser was used as the excitation source, with a 50× objective lens, a signal acquisition time of 30 s, and two accumulations per measurement. The grating was set to 600 lines/mm, with a slit width of 300 μm and an instrument resolution of 2 cm^−1^. Calibration was performed using a monocrystalline silicon wafer. The spectral range covered 100–4000 cm^−1^.

#### 2.2.4. Fourier-Transform Infrared Spectroscopy (FTIR)

FTIR analysis was performed on GB-03 (oil-coated ground layer) and GB-13 (polychrome ground layer) to identify both inorganic mineral components and organic functional groups associated with the binding media. Infrared spectra were acquired using a Nicolet IS50 Fourier-transform infrared spectrometer (Thermo Fisher, Waltham, MA, USA) equipped with a diamond crystal attenuated total reflectance accessory. The spectra were recorded at a resolution of 4 cm^−1^ over a wavenumber range of 400–4000 cm^−1^.

#### 2.2.5. X-Ray Diffraction

XRD analysis was performed on two ground-layer samples: GB-03 (oil-coated ground layer) and GB-13 (polychrome ground layer), to identify their inorganic mineral components. X-ray diffraction analysis was conducted using an Ultima IV multifunctional X-ray diffractometer (Rigak, Tokyo, Japan). The operating conditions were as follows: Cu Kα radiation (λ = 1.5406 Å), tube voltage 40 kV, tube current 40 mA, with a Ni filter as monochromator and a D/teX-Ultra high-speed detector (Rigak, Tokyo, Japan). Data were collected in θ/2θ continuous scanning mode over a 2θ range of 3–70°, with a scanning speed of 8° per minute and a step width of 0.02°. Semi-quantitative phase abundances were determined via the reference intensity ratio (RIR) method, by normalizing the ratios of each phase’s strongest peak intensity to its PDF-2 RIR value.

#### 2.2.6. Pyrolysis-Gas Chromatography/Mass Spectrometry(Py-GC/MS)

Py-GC/MS analysis was performed on GB-03 (oil-coated ground layer) and GB-13 (polychrome ground layer) to identify their organic binding media. Approximately 0.2 mg of each sample was placed in a pyrolytic sample cup and introduced into the pyrolysis unit. Pyrolysis was performed using an EGA/PY-3030D tube furnace pyrolyzer (Frontier Lab, Fukushima, Japan) at 550 °C, coupled to a gas chromatography–mass spectrometry system (Agilent 8860-5977B (Agilent, Santa Clara, CA, USA), with Frontier injection). The chromatographic conditions were as follows: carrier gas high-purity helium in constant flow mode; split ratio 20:1; separation column UltraALLOY+-5 (Frontier Lab, 30 m × 0.25 mm × 0.25 μm film thickness, stationary phase 5% phenyl-95% methylpolysiloxane). The pyrolysis temperature was maintained at 550 °C for 12 s. The injector temperature was set to 280 °C, and the interface temperature between the injector and the chromatograph was 300 °C. The column oven temperature program began at 50 °C, held for 5 min, then ramped to 300 °C at 5 °C/min and held for 10 min. Mass spectrometric detection was performed with a quadrupole mass analyzer. The quadrupole temperature was set to 150 °C, and the MS transfer line temperature was 320 °C. Electron ionization was employed at an ion source voltage of 70 eV and an ion source temperature of 230 °C. Full-scan mode was applied over a mass range of m/z 29–600, with a solvent delay of 1 min. The resulting data were processed using MassHunter Unknowns Analysis software (version 10.1), and spectral identification was performed by comparison with the NIST17 mass spectral library.

## 3. Results

### 3.1. Cross-Sectional Microscopy

**Figure 1:**
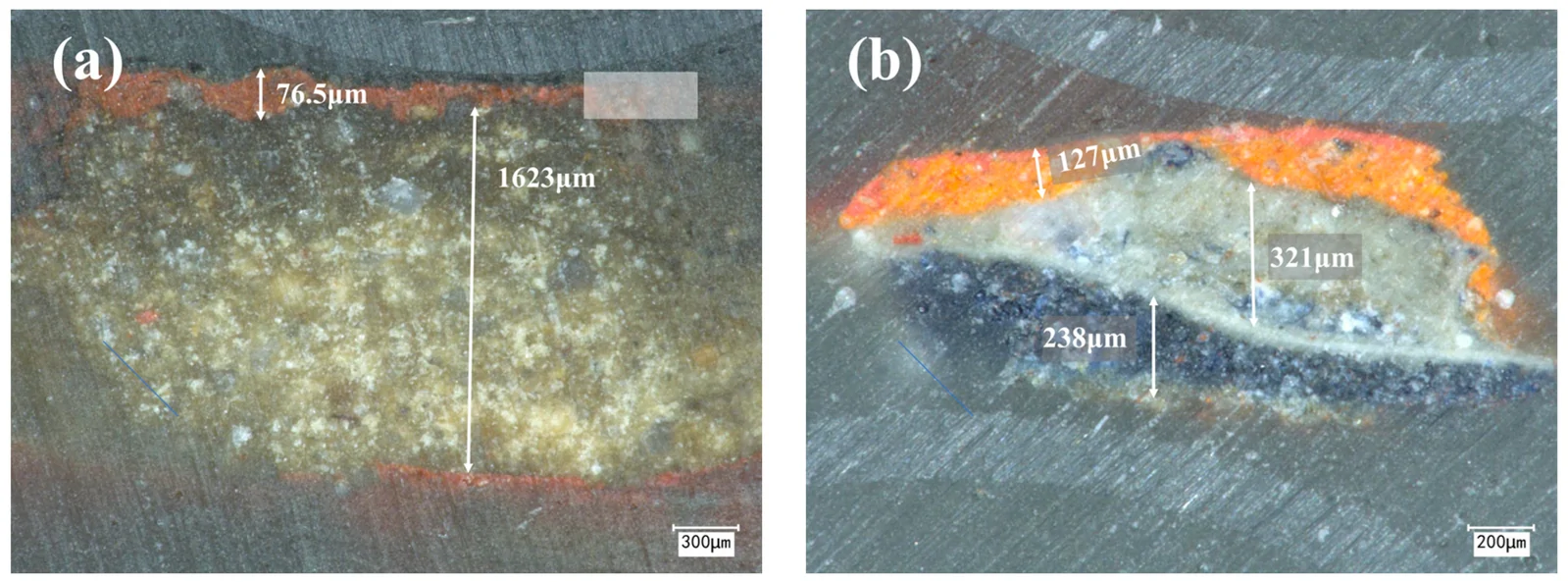
Cross-sectional micrographs of representative samples: (**a**) GB-03; (**b**) GB-13.

Figure 1 presents the sampling photographs and cross-sectional structures of the polychrome and oil-coated samples. Sample GB-03 reveals that the oil coating on the stele pavilion was repainted. The initial oil coating was red and applied directly onto the column surface, with a thickness of approximately 76.5 μm. At a later stage, a single-layer ash ground layer, approximately 1623 μm thick, was applied over the red oil coating. This ground layer appears light brown and contains dispersed white and gray mineral particles. A new red oil coating was subsequently applied over this ground layer (Figure 1a).

The polychrome paintings on the stele pavilion generally employ a single-layer ash ground. Due to prolonged outdoor exposure, severe powdering of the paint layers has occurred. Sample GB-13 exhibits a distinctive technological feature: it is a multilayered repainted structure comprising a red surface layer and a blue lower layer. The red paint layer was applied over an orange underlayer, and the combined thickness of the orange and red layers is approximately 127 μm. A small amount of blue pigment is present beneath the orange layer. Between the blue surface layer and the underlying blue layer, a white ground layer approximately 321 μm thick serves as a separator. The lower blue pigment layer measures approximately 238 μm in thickness (Figure 1b).

### 3.2. Raman Spectroscopy

**Figure 2:**
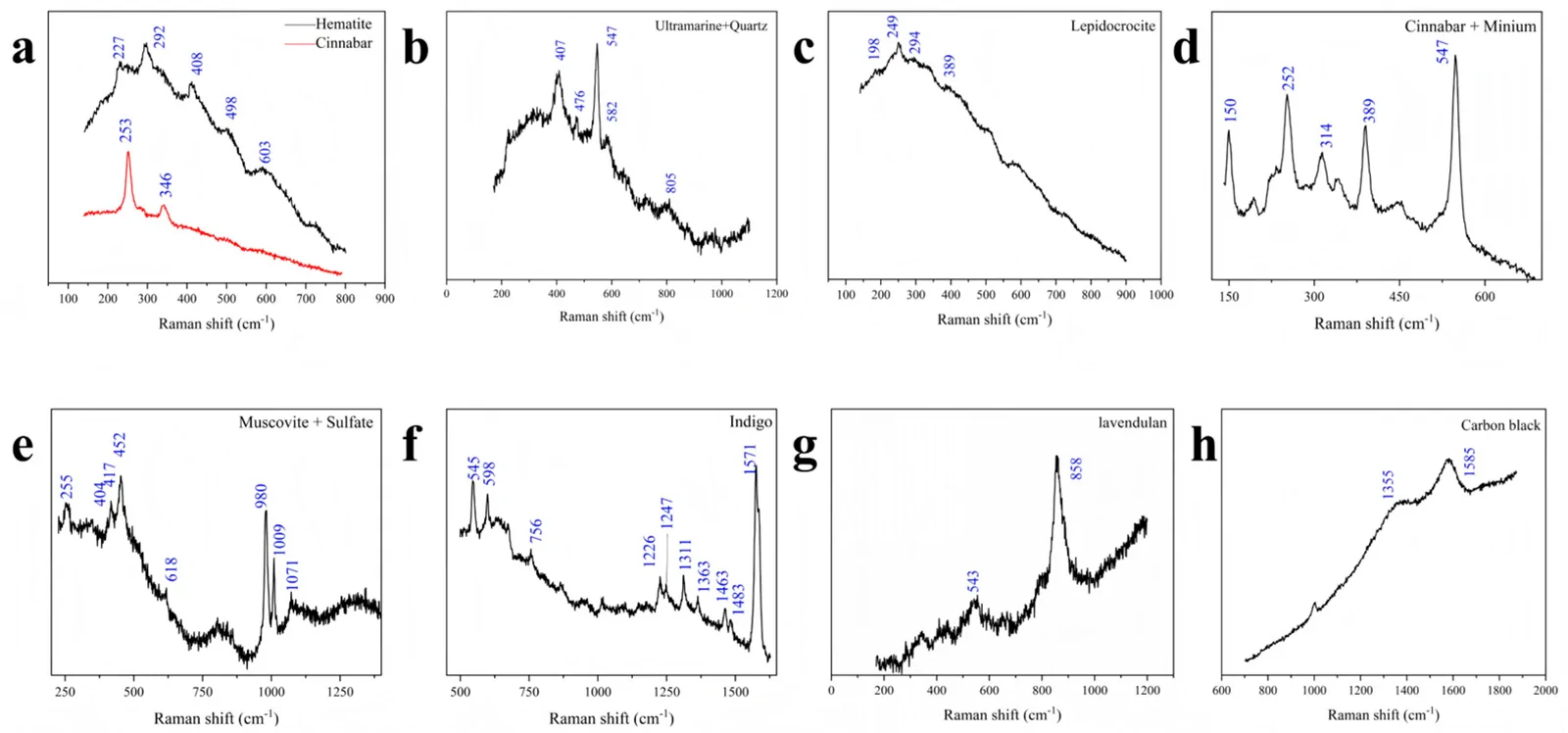
Raman spectra of pigments from different-colored samples: (**a**) GB-03; (**b**) GB-07; (**c**) GB-09; (**d**) GB-13 (Cinnabar + Minium); (**e**) GB-13 (Muscovite + Sulfate); (**f**) GB-13 (Indigo); (**g**) GB-15; (**h**) GB-17.

The Raman spectra of the different-colored pigments are shown in Figure 2. For the oil-coated sample GB-03, the characteristic peaks of the red surface pigment at 227, 292, 408, 498, and 603 cm^−1^ correspond to hematite (iron red), whereas the red underlying pigment exhibits peaks at 253 and 346 cm^−1^, indicative of cinnabar.

The blue pigment in sample GB-07 displays characteristic peaks at 547 and 582 cm^−1^, attributed to the symmetric stretching vibrations of S_2_^−^ and S_3_^−^ radicals, confirming the pigment as ultramarine. Additional peaks at 407 and 474 cm^−1^ correspond to quartz.

Sample GB-09 shows Raman peaks at 198, 249, 294, and 389 cm^−1^, consistent with the standard Raman spectrum of lepidocrocite (γ-FeOOH). Lepidocrocite is an iron oxyhydroxide that forms a polymorphic pair with goethite (α-FeOOH) and is commonly associated with it in nature. The chromophoric mineral in this yellow pigment can therefore be identified as lepidocrocite.

For sample GB-13, the Raman spectrum of the upper pigment layer exhibits peaks at 150, 252, 314, 389, 447, and 547 cm^−1^, indicating a mixture of cinnabar and red lead. The lower blue pigment shows characteristic peaks at 545, 598, 756, 1226, 1247, 1311, 1363, 1463, 1483, and 1571 cm^−1^, corresponding to indigo. Additionally, typical triple peaks at 417, 452, and 497 cm^−1^ are assignable to mica-group minerals, indicating that the inorganic matrix is predominantly composed of layered silicates. Peaks at 980 and 1009 cm^−1^ indicate the presence of sulfate ions, suggesting that the pigment consists of a mixture of indigo and mica [19].

Sample GB-15 exhibits its strongest peak at 858 cm^−1^, attributable to the symmetric stretching vibration (ν_1_) of AsO_4_^3−^, and a strong peak at 540 cm^−1^ assignable to the antisymmetric bending vibration (ν_4_) of AsO_4_^3−^. This green pigment is thus identified as lavendulan.

Sample GB-17 shows characteristic peaks at 1330 and 1592 cm^−1^, confirming the presence of carbon black.

### 3.3. SEM-EDS

**Figure 3:**
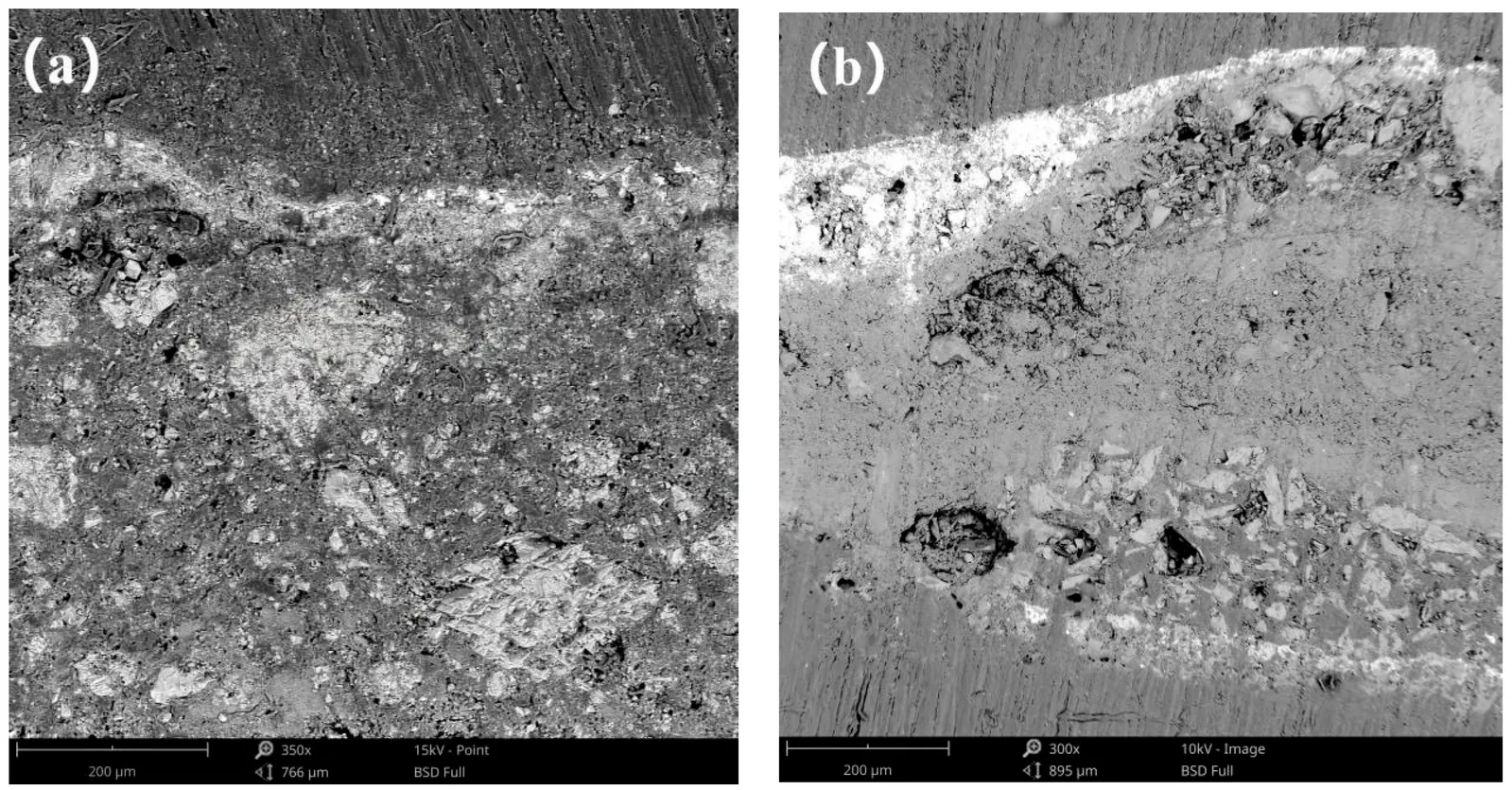
Backscattered scanning electron micrographs: (**a**) GB-03 oil-coated ground layer; (**b**) GB-13 multi-layered polychrome.

To determine the elemental composition of the ground layer materials, energy-dispersive spectroscopic analysis was performed on different pigment particles. Table 2 summarizes the EDS results for selected polychrome samples, and Figure 3 presents backscattered electron micrographs of representative samples.

Under scanning electron microscopy, the GB-03 oil-coated ground layer sample reveals large white particles (Figure 3a) containing exclusively calcium, identified as calcium carbonate particles. The surrounding gray areas contain, in addition to calcium, minor amounts of silicon, magnesium, sodium, and aluminum, suggesting the presence of other clay-type materials.

EDS analysis of the gold leaf on sample GB-05 detected Au (99.48%), Cu (0.33%), and Ag (0.19%), confirming the use of leaf gold.

For sample GB-07, four distinct blue particles were selected under optical microscopy (Figure S1) for scanning electron microscopy observation (Figure S2). The pigment particles exhibit a certain degree of agglomeration. These agglomerated particles are composed of fine crystals with sub-rounded to irregular morphology. The overall size of the agglomerated particles ranges from approximately 5 to 20 μm, showing variability in particle dimensions. This morphological feature is consistent with that of synthetic ultramarine reported in the literature [20]. Polarized light microscopy (Figure S3) further reveals that the blue particles appear sub-rounded with similar sizes and exhibit a deep blue coloration. Energy-dispersive X-ray spectroscopy was performed on these four blue particles. The results show that sodium, aluminum, silicon, and sulfur are the principal elements. Semi-quantitative analysis indicates that the Na/S atomic ratio is consistently below 1 for all measured spots [21]. This ratio serves as a diagnostic characteristic of synthetic ultramarine. The combined evidence of particle morphology, Na/S ratio, and the absence of accessory minerals characteristic of natural lapis lazuli as determined by Raman spectroscopy leads to the identification of sample GB-07 as synthetic ultramarine rather than natural lapis lazuli.

Sample GB-09 (yellow) contains Fe, which, in combination with the Raman results, confirms the yellow pigment as iron yellow.

Sample GB-13 (multilayered repainted polychrome) shows that the orange-red pigment layer contains Hg, Pb, and S, indicating a mixture of cinnabar and red lead. Beneath the orange-red layer lies a blue pigment layer (Figure 3b) with the same elemental composition as the lower blue layer. Given the Raman detection of indigo and mica, together with the high potassium content, the blue pigment is inferred to be indigo mixed with mica. The white ground layer between the two blue layers contains Al, Si, and O, consistent with kaolinite as the ground material.

Sample GB-15 contains As, Cu, Na, and Cl. In combination with Raman results, the green material is identified as lavendulan.

### 3.4. X-Ray Diffraction and FTIR

The ground layers are classified into oil-coated ground layers and polychrome ground layers. Different materials were used in these layers depending on their location. Infrared spectroscopy and X-ray diffraction confirmed the phase differences.

**Figure 4:**
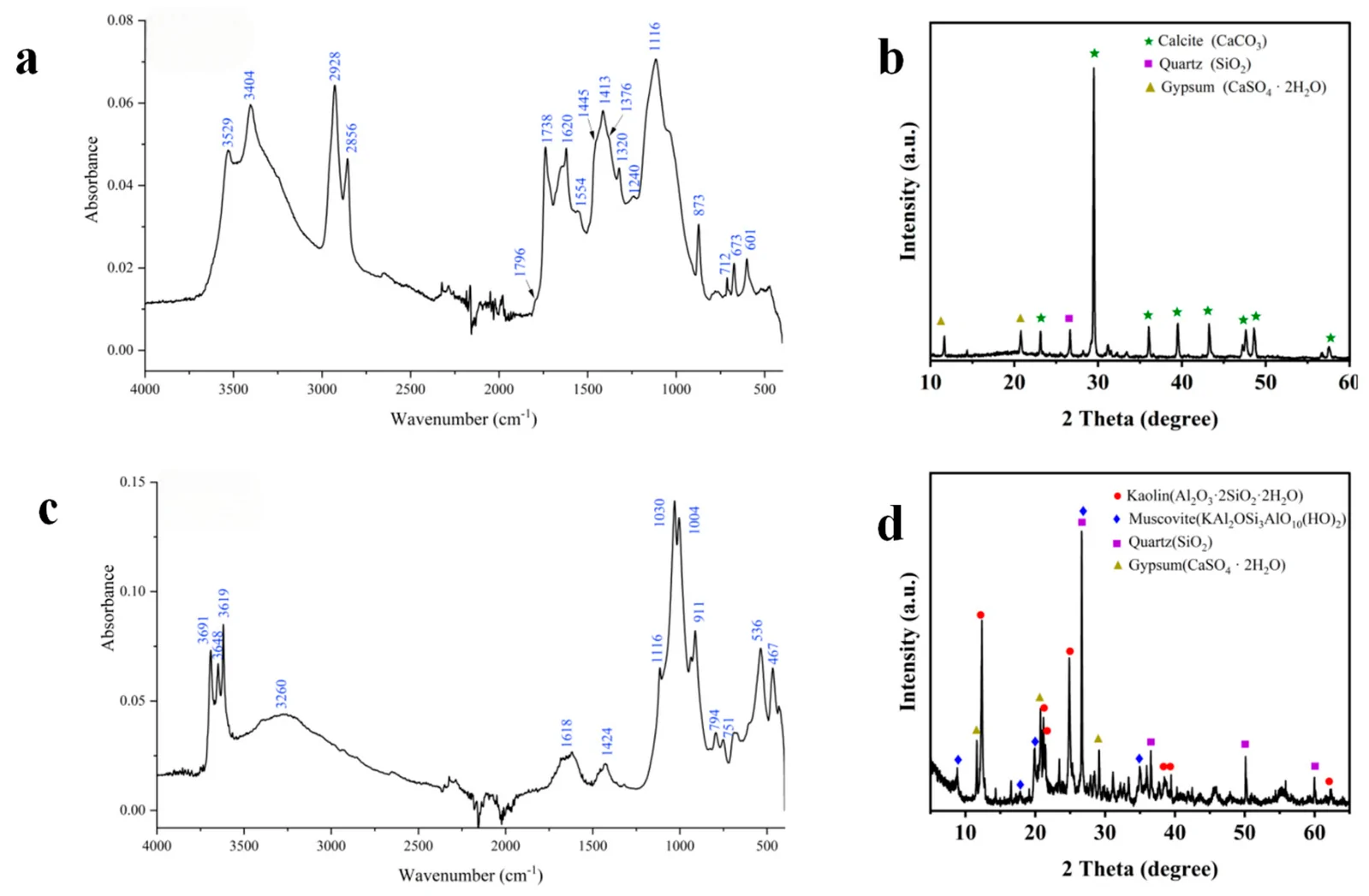
Phase analysis of ground layers: (**a**) FT-IR spectrum of oil-coated ground layer; (**b**) XRD pattern of oil-coated ground layer; (**c**) FT-IR spectrum of polychrome ground layer; (**d**) XRD pattern of polychrome ground layer.

The infrared spectrum of the oil-coated ground layer sample is presented in Figure 4a. It exhibits several characteristic absorption bands. The peaks at 2928 and 2856 cm^−1^ are assigned to the antisymmetric and symmetric stretching vibrations of CH_2_ groups. Another peak at 1738 cm^−1^ corresponds to the C=O carbonyl stretching vibration. These features confirm the presence of substantial lipid components. Additional peaks indicate abundant calcium carbonate. These include the antisymmetric stretching of CO_3_^2−^ at 1445 cm^−1^, the out-of-plane bending at 873 cm^−1^, and the in-plane bending at 712 cm^−1^. The band at 1116 cm^−1^ arises from the O-Si-O antisymmetric stretching. The peak at 673 cm^−1^ is attributable to the OH wagging vibration of gypsum, while the peak at 601 cm^−1^ corresponds to the out-of-plane bending of SO_4_^2−^. Other observed bands include the H-O-H bending of gypsum hydration water at 1620 cm^−1^ and the symmetrical deformation of methyl groups in organic matter at 1376 cm^−1^ (symmetrical deformation of methyl groups in organic matter). The band near 1320 cm^−1^ is tentatively assigned to C-H bending and C-C skeletal vibrations.

X-ray diffraction analysis (Figure 4b) confirms that the principal components are calcite, quartz, and gypsum. The semi-quantitative XRD results (Table 3) indicate that the oil-coated ground layer consists of 86% calcite, 6% quartz, and 7% gypsum.

The infrared spectrum of the polychrome ground layer (Figure 4c) exhibits sharp absorption peaks at 3691, 3648, and 3619 cm^−1^, attributable to the stretching vibrations of structural hydroxyl groups (OH) in kaolinite-group layered silicate minerals, indicating well-crystallized kaolinite. The peak near 1618 cm^−1^ corresponds to the bending vibration of OH. The peaks at 938 and 911 cm^−1^ are assignable to the bending vibrations of internal OH and inner-surface OH, respectively. The peaks at 1116 and 1030 cm^−1^ correspond to O-Si-O antisymmetric stretching, while 1004 cm^−1^ appears as a shoulder of Si-O stretching. The peak at 794 cm^−1^ is attributed to O-Si-O symmetric stretching, 536 cm^−1^ to Si-O-Al bending, and 467 cm^−1^ to Si-O-Mg absorption. These features indicate that the ground layer filler contains well-crystallized, high-purity clay minerals.

X-ray diffraction analysis (Figure 4d) confirms that the principal components are quartz, gypsum, kaolinite, and minor mica, consistent with the infrared results. The semi-quantitative XRD results (Table 3) indicate that the polychrome ground layer consists of 35% quartz, 12% gypsum, 7% mica, and 47% kaolinite.

### 3.5. Py-GC/MS

**Figure 5:**
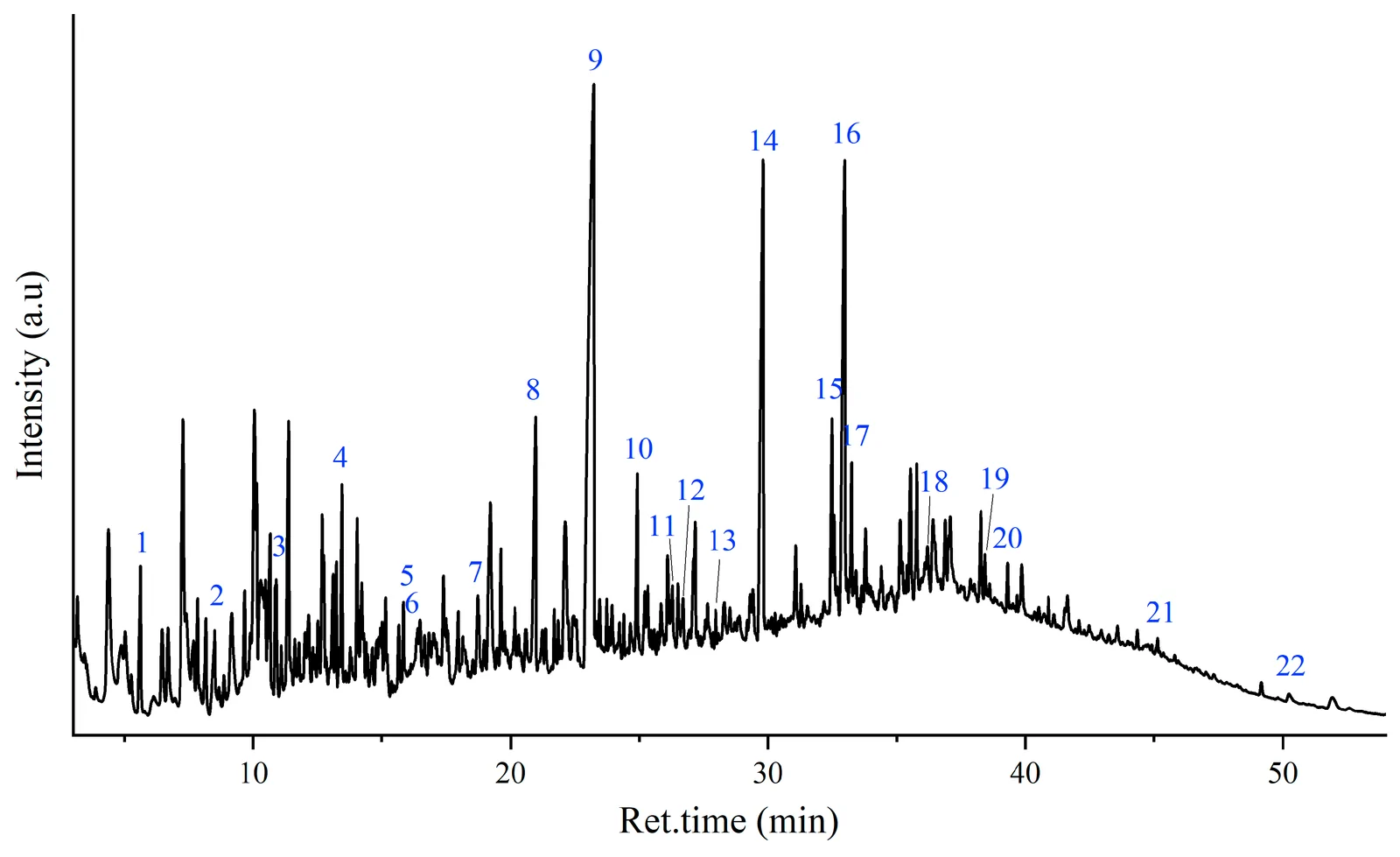
Total ion current chromatogram (TIC) of the oil-coated ground layer sample (GB-03) analyzed by Py-GC/MS with thermally assisted methylation. Peak numbers correspond to the compounds listed in Table 4.

**Figure 6:**
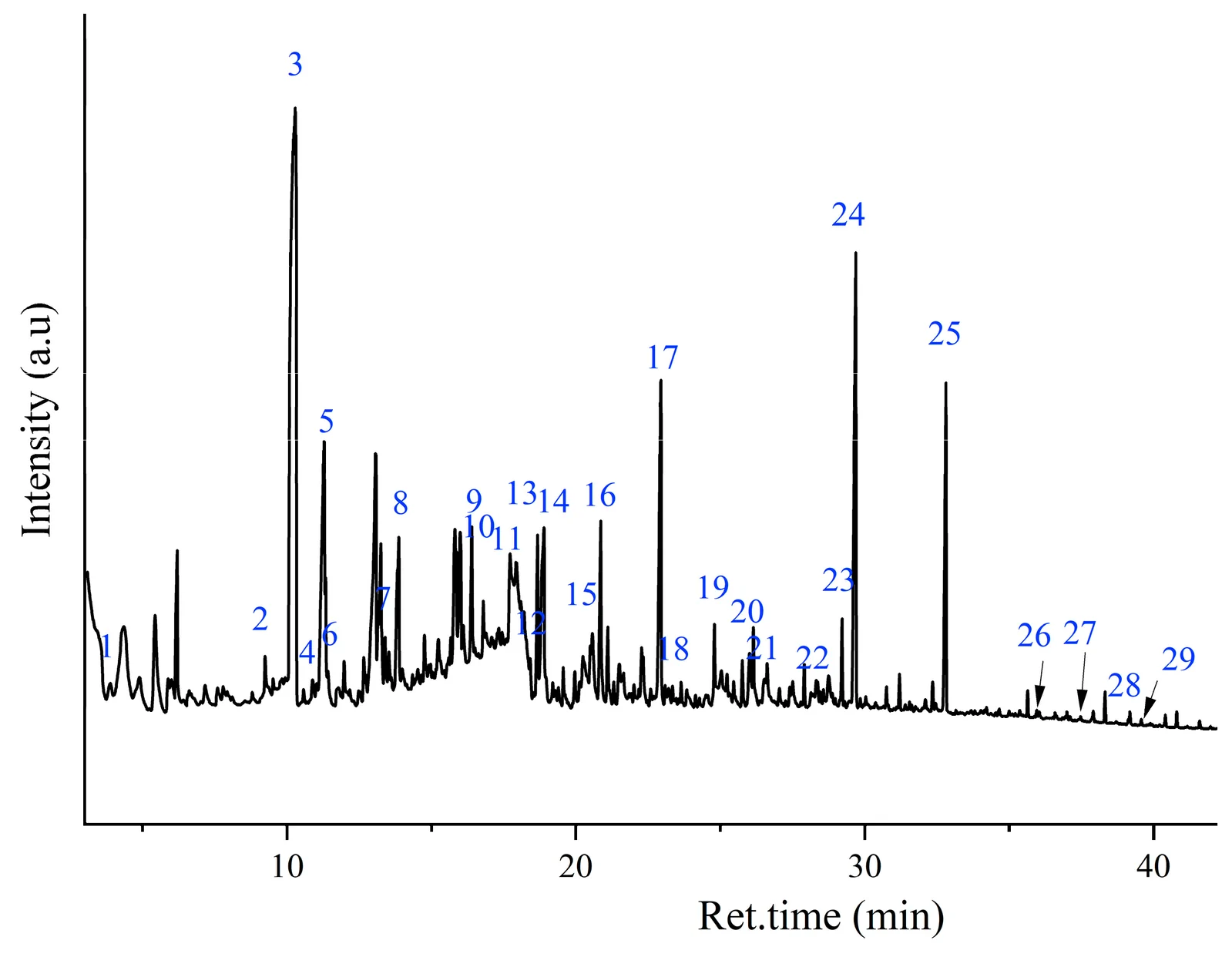
Total ion current chromatogram (TIC) of the polychrome painting sample (GB-13) analyzed by Py-GC/MS with thermally assisted methylation. Peak numbers correspond to the compounds listed in Table 5.

To investigate the organic binding media in the ground layers, pyrolysis-gas chromatography/mass spectrometry was employed. The total ion current chromatograms of the oil-coated ground layer and the polychrome painting sample are shown in Figure 5 and Figure 6, respectively. Table 4 and Table 5 list the characteristic pyrolysis products detected in each sample.

#### 3.5.1. Oil-Coated Ground Layer (GB-03)

The oil-coated ground layer sample analyzed by thermally assisted methylation GC/MS revealed abundant monocarboxylic acids, dicarboxylic acids, and alkylphenyl alkanoate (APAS) markers characteristic of heat-bodied oils. Particularly noteworthy is the detection of a substantial amount of dimethyl azelate (#9, 31.03%), a typical oxidation product of drying oils. The P/S ratio (palmitic acid/stearic acid) was calculated as 1.13, and the A/P ratio (azelaic acid/palmitic acid) as 1.4, indicating that the primary organic binder in the oil-coated ground layer is heat-bodied tung oil.

The detection of methyl dehydroabietate (#18) and methyl 7-oxodehydroabietate (#20) further confirms the presence of rosin resin in the oil-coated ground layer. The presence of methyl behenate (#19) and phytosterol derivatives (#21 and 22) is consistent with the use of vegetable oils.

#### 3.5.2. The Polychrome Painting (GB-13)

The polychrome painting sample analyzed by thermally assisted methylation GC/MS revealed pyrrole derivatives, indole derivatives, proline derivatives, and trimethyl phosphate. Pyrrole derivatives (#1, 4, and 7) are typical pyrolysis products of animal glue, while indole derivatives (peaks 11, 18, and 22), proline derivatives (#6, 14, and 15), trimethyl phosphate (#2), blood/egg white marker 5 (#12), and cholesterol derivatives (#29) are characteristic of pig blood [22,23]. The proteinaceous components in the polychrome ground layer are therefore identified as a combination of animal glue and pig blood.

The polychrome ground layer sample also contains abundant monocarboxylic and dicarboxylic acids. The monocarboxylic acids are predominantly palmitic acid (C16) and stearic acid (C18), detected as their methyl esters (#24 and 25). The presence of dimethyl azelate (#17, 6.79%), a characteristic oxidation product of drying oils, indicates the inclusion of a substantial drying oil component in the polychrome ground layer. No APAS markers were detected, suggesting that this drying oil was not heat-bodied and was applied in its raw, unheated state. Although animal glue and pig blood can contribute minor fatty acid content, their contribution is far below the levels detected in this sample; the drying oil is therefore inferred to have been deliberately added to the polychrome ground layer.

Additionally, methyl dehydroabietate (#26), methyl-7-methoxytetrahydroabietate (#27), and methyl 7-oxodehydroabietate (#28) were detected, confirming the presence of rosin resin in the polychrome ground layer.

Figure 6 shows total ion current chromatogram (TIC) of the polychrome painting sample (GB-13) analyzed by Py-GC/MS with thermally assisted methylation. Peak numbers correspond to the compounds listed in Table 5.

## 4. Discussion

### 4.1. Pigment

Prior to the late Qing Dynasty, mineral pigments predominated in architectural polychrome paintings. From the mid-Ming to the early Qing, polychrome paintings in the Jiangnan region typically employed cinnabar, red lead, chalk, lead white, atacamite, malachite, azurite, indigo, and yellow earth [24]. During the mid-Qing, the polychrome paintings in Baoguang Hall of Prince Gong’s Mansion incorporated azurite, indigo, Prussian blue, lapis lazuli, synthetic ultramarine, smalt, basic copper chloride, vermilion, red lead, hematite, orpiment, chalk, lead white, kaolin, and carbon black [25]. The pigments used in Shanxi regional polychrome paintings have also been documented as predominantly traditional mineral materials [14]. The pigments identified in this study—hematite, cinnabar, red lead, iron yellow, ultramarine, indigo, and carbon black—are all consistent with the commonly used palette of Qing-dynasty architectural decoration.

The red oil coating on the stele pavilion exhibits evidence of repainting. Two distinct red layers are separated by an intervening ground layer, indicating two independent construction phases. The underlying red pigment was identified as cinnabar, whereas the surface layer consists of hematite. Although the Qing-dynasty official construction manuals, such as *Gongbu Gongcheng Zuofa Zeli* (the Engineering Methods of the Ministry of Works) and *Neiting Gongcheng Zuofa* (the Imperial Engineering Method), were codified for official architecture, their records of pigment prices and technological specifications still provide useful reference points for understanding material choices in regional buildings. According to Liu’s compilation [1] of pigment valuations from these manuals, the price differential between cinnabar and red earth was substantial. *Jiuqing Yiding Wuliao Jiazhi* (The Deliberated Material Values of the Nine Ministers) records that arrow-tip cinnabar cost 4 taels per catty (jin, approx. 600 g under the Qing treasury standard), whereas top-grade red earth was valued at merely 0.014 tael (1 fen 4 li) per catty. Even synthetic vermilion, priced between 0.14 and 0.5 tael (1 qian 4 fen and 5 qian) per catty, remained substantially more expensive than red earth. The stele pavilion was erected in the mid-Qing to commemorate Prince Guoqin’s poetry stele. Given its status as a local Daoist building, the original use of cinnabar is plausible. This pigment was also commonly employed in official architecture. The later repainting, which involved a newly applied ground layer and hematite-based oil coating, was likely executed after the late Qing, a period when Shanxi’s regional economy had generally declined. Under such circumstances, community-funded restoration projects tended to favor lower-cost materials, and the substitution of hematite for cinnabar aligns with this economically rational choice. However, this interpretation remains speculative in the absence of direct documentary or chronological evidence linking this specific repainting campaign to a particular historical period.

The green pigment in sample GB-15 was identified as lavendulan (NaCaCu_5_(AsO_4_)_4_Cl·5H_2_O), a copper arsenate mineral. In the field of cultural heritage conservation, this mineral is most frequently reported as a degradation product of other pigments. A review of the recent literature reveals at least two well-documented formation pathways, originating respectively from emerald green and atacamite. Li et al. [26] demonstrated that during the degradation of emerald green in the polychrome sculptures of Dazu Rock Carvings in Chongqing, arsenite ions (AsO_2_^−^) were oxidized to arsenate (AsO_4_^3−^), which subsequently combined with Cu^2+^ in the presence of Na^+^, Ca^2+^, and Cl^−^ from groundwater to form blue-green lavendulan. Shen et al. [27] identified an independent transformation pathway in the Kizil Grotto murals in Xinjiang, where atacamite (Cu_2_(OH)_3_Cl) was converted in situ into lavendulan under the influence of AsO_4_^3−^, Na^+^, and Ca^2+^. In the present sample, neither emerald green nor atacamite was detected as a precursor. The specific origin of lavendulan in this sample therefore remains uncertain, and both possible pathways cannot be excluded.

The blue pigment in polychrome sample GB-07 was identified as synthetic ultramarine. Lapis lazuli was one of the primary sources of natural blue mineral pigments and was widely employed in cave murals and polychrome artworks along the Silk Road [28]. However, the rarity of natural lapis lazuli and the labor-intensive extraction process led to the industrial synthesis of ultramarine in France in 1828. This synthetic pigment was introduced to China only after the mid-19th century [1]. It has since been widely used in wall paintings [29] and architectural polychrome decorations [30]. The identification of synthetic ultramarine in GB-07 therefore indicates that this paint layer was applied no earlier than the mid-19th century, providing a terminus post quem for this section of the polychrome painting.

Cross-sectional analysis of polychrome sample GB-13 revealed a stratigraphic sequence of red-orange-blue-white-blue, confirming that the stele pavilion underwent at least one repainting campaign. The red layer consists of a traditional formulation: a red lead underlayer overpainted with cinnabar. The blue pigment was identified as indigo mixed with mica. A kaolinite ground layer separates the two blue layers. Indigo has a long history of use in Chinese architectural polychromes and has been documented in other buildings, including the polychrome paintings of Jianfu Palace [31] and Xianfu Palace [32]. The mixture of indigo with mica, observed in the third and fifth layers of GB-13, has a well-documented historical precedent in Qing-dynasty polychrome painting. Scientific analysis of the Qing mid-period paste-paper pigments from Suichu Hall in the Ningshou Palace complex of the Forbidden City revealed that light blue ground colors contained indigo and mica, with the mica serving to impart a sparkling luster effect to the pigment [33]. Notably, EDS analysis of the blue layers revealed a measurable arsenic content, and the corresponding Raman spectrum displayed a broad hump near 812 cm^−1^ without sharp characteristic peaks of crystalline arsenates, suggesting that arsenic is present in a low-crystallinity or adsorbed state. Given the detection of lavendulan in GB-15, it is plausible that this arsenic signal resulted from the migration of arsenate species within the painted structure.

The yellow pigment was identified as iron yellow, a clay-based pigment composed primarily of iron oxides and hydroxides. Its mineral phase is predominantly goethite (α-FeOOH), often associated with hematite and may contain impurities such as quartz and clay minerals. Iron-containing yellow pigments have a long history of use worldwide and have been identified in the polychrome paintings of Kaihua Temple’s Great Hero Hall [34] and in the murals of Zhulong Temple in Yu County, Shanxi, from the Qing Dynasty [35].

EDS analysis of the gold leaf on the carved dragon bracket (sample GB-05) revealed a gold content of 99.48%, together with minor copper (0.33%) and silver (0.19%). This purity level conforms to the traditional standard of “ku jin” (palace-grade gold leaf). In Qing-dynasty official architectural practice, ku jin represented the highest grade of gold leaf, with gold content typically ranging between 95% and 98% [1], and was reserved for the wood carvings, dragon motifs, and high-ranking polychrome components of major buildings. The stele pavilion houses a poetry stele inscribed in 1734 by Prince Guoqin during his visit to the Guandi Temple. The presence of this imperial stele endowed the pavilion with symbolic significance beyond that of an ordinary pavilion structure. It served both as a material testament to imperial veneration of Guan Yu’s martial loyalty and as a marker of the building’s elevated status within the Guandi Temple complex. The use of high-purity ku jin in the pavilion’s polychrome decoration is likely related to this special status.

### 4.2. Ground Layers

According to historical records, traditional Shanxi polychrome painting employed four types of ground preparation techniques: oil-ash ground, blood-based putty ground, alum-glue-ground, and loess-flour ground. In Shanxi regional practice, ground preparation refers specifically to the first or foundational plaster/putty layer applied directly onto the wooden substrate [36]. The polychrome ground layer of the stele pavilion’s late-Qing polychrome paintings was found to contain quartz, gypsum, kaolinite, and minor mica, with kaolinite as the dominant component. Kaolinite particles are fine in size and exhibit good plasticity and high whiteness, providing a smooth, dense, and light-colored foundation for the polychrome layer. Moreover, the high chemical activity and adsorption capacity of kaolinite particle surfaces facilitate effective bonding with mineral pigments dispersed in proteinaceous media.

Pyrolysis-gas chromatography/mass spectrometry of the polychrome ground layer sample revealed the presence of drying oil, rosin, animal glue, and pig blood. Historical records indicate that traditional Shanxi ground preparation techniques employing pig blood and tung oil as binding materials are referred to as tung oil-pig blood ground techniques [37]. The drying oil, rosin, and blood-derived materials detected in this sample are therefore interpreted as the binding media of the polychrome ground layer, whereas the animal glue is attributed to the binder used for pigment dispersion in the paint layer itself.

The oil-coated ground layer of the stele pavilion is characterized by calcite as the dominant aggregate at 83%. The high calcite content would have formed a hard, dense substrate capable of ensuring the smoothness and durability of the overlying oil coating. Combined with in situ observations and cross-sectional microscopy, no hemp or other fibrous materials were detected in the ground layer, indicating that the ground preparation technique was a single-layer ash application.

The organic binders in the oil-coated ground layer were identified as heat-bodied tung oil combined with rosin. Heat-bodied tung oil serves to waterproof the ground layer, resist decay and insect infestation, and thereby enhance overall ground layer performance. The combination of tung oil and rosin is commonly encountered in oil-coated ground layers. Similar formulations have been detected in the ground layers of Jide Hall’s architrave [38], the oil-coated ground of Wenyuan Hall in the Forbidden City [39], and in door and window lacquer layers [40] as well as relief gold-leaf applications [41]. The addition of rosin during the boiling of tung oil accelerates the drying process of the oil while simultaneously improving its adhesion and gloss. In the context of traditional Shanxi polychrome painting techniques, this type of ground formulation differs from other indigenous Shanxi ground preparation methods. Given that this ground layer was a later reapplication, it does not appear to have adhered to the standard techniques of Qing-dynasty Shanxi regional practice. Instead, it represents a simplified formulation, constituting a localized technological variant or an atypical recipe.

## 5. Conclusions

This study presents the first systematic scientific analysis of the polychrome paintings and oil coatings on the stele pavilion of the Guandi Temple in Shanxi, employing a comprehensive array of analytical techniques to elucidate their material composition and technological characteristics of these decorative layers.

The pigment palette identified in the stele pavilion is remarkably diverse, encompassing hematite, cinnabar, red lead, ultramarine, indigo, iron yellow, lavendulan, and carbon black. The gilded dragon carving was executed with high-purity palace-grade gold leaf. The oil coating exhibits evidence of repainting following the reapplication of the ground layer: the original oil coating was formulated with cinnabar, whereas the later repainting employed hematite as the red pigment. The polychrome painting samples reveal multilayered repainting sequences, with the surface layer composed of a mixture of cinnabar and red lead and the lower layer consisting of indigo mixed with mica, separated by a kaolinite ground layer.

The technological features of the oil-coated ground layer and the polychrome ground layer differ considerably. The reapplied oil-coated ground layer was executed as a single-layer ash preparation, with calcite, quartz, and gypsum as the inorganic fillers, and heat-bodied tung oil combined with rosin as the organic binder. The polychrome ground layer is dominated by kaolinite, quartz, and gypsum, with the organic binder comprising a mixture of animal glue and pig blood, supplemented by unheated drying oil and rosin.

This study represents the first scientific investigation of the polychrome paintings on the Guandi Temple stele pavilion. Severe powdering of the paint layers was observed. The detection of lavendulan in the green pigment, together with the presence of arsenic in other pigment layers from the same pavilion, raises the possibility of arsenate mobilization as a potential contributor to paint layer deterioration. These findings not only provide new material evidence for the regional technological characteristics of Qing-dynasty polychrome painting in Shanxi, but also contribute scientific data for understanding the degradation mechanisms of Qing-dynasty polychrome decorations. Furthermore, this study provides a methodological reference for the investigation and conservation of Ming-Qing architectural polychrome paintings in northern China.

The present findings should be regarded primarily as a site-specific analytical case study. Broader generalizations about the technological characteristics of Qing-dynasty regional architectural painting in Shanxi will require comparative analyses of additional buildings and larger sample sets. The proposed arsenate migration mechanism remains a hypothesis, suggested only by the co-occurrence of arsenic in multiple samples. Future work employing higher-resolution spatial analytical techniques, such as synchrotron-based micro-XRF mapping, will be essential to test this hypothesis and to elucidate the pathways and extent of arsenate mobilization and its role in paint layer deterioration.

## Figures and Tables

**Table 1:** Sampling Information of Color Paintings from the Stele Pavilion of Guandi Temple in Shanxi.

Sample ID	Name	Color	Photograph
GB-03	Column oil coating	Red	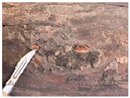
GB-05	Carved dragon on architrave	Gold	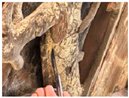
GB-07	Carved architrave	Blue	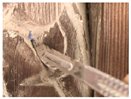
GB-09	Carved dragon on architrave	Yellow	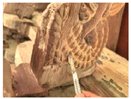
GB-13	Floating-cloud architrave	Red and blue	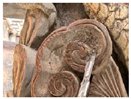
GB-15	Eaves purlin painting	Green	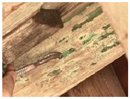
GB-17	Architrave painting	Black	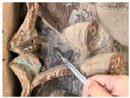

**Table 2:** SEM-EDS analysis results of selected polychrome painting samples.

Sample	Spot	Detected Elements (wt%)	Identified Material
GB-03	Red surface	O (69.94), Fe (30.06)	Hematite (iron red)
	Red underlying	Hg (86.22), S (13.78)	Cinnabar
	Ground (bright particles)	C (42.37), O (31.70), Ca (26.02)	Calcium carbonate
	Ground (matrix)	C (44.33), Ca (28.46), O (25.33), Si (0.72), Mg (0.41), Na (0.40), Al (0.35)	Calcium carbonate + clay minerals
GB-05	Gold leaf	Au (99.48), Cu (0.33), Ag (0.19)	Leaf gold
GB-07	Blue	O(33.66), Si(23.61), N(8.57), Al(12.14), S(13.35), Na(7.26), K(0.62)	synthetic ultramarine
GB-09	Yellow	O (55.26), Si (18.30), Al (14.27), Fe (6.18), K (2.91), S (2.26), Ca (0.82)	Lepidocrocite (iron yellow)
GB-13	Orange-red	O (54.63), Pb (29.90), Hg (13.62), S (1.85)	Cinnabar + red lead
	Blue (upper)	Si (34.58), O (13.89), N (29.19), K (8.87), Al (9.06), As (1.31), Na (0.67)	Indigo + mica
	White ground	O (76.88), Si (11.60), Al (11.52)	Kaolinite
	Blue (lower)	Si (40.28), O (28.40), N (16.98), K (7.41), Al (4.41), As (2.07), Na (0.45)	Indigo + mica
GB-015	Green	As (33.13), O (31.29), Cu (23.65), Na (6.22), Cl (4.38), Ca (1.33)	lavendulan

**Table 3:** XRD analysis results of inorganic components (%).

Sample	Calcite	Quartz	Gypsum	Mica	Kaolinite
GB-03	86	6	7	-	-
GB-13	-	35	12	7	47

Note: “-” indicates not detected.

**Table 4:** Characteristic pyrolysis products detected in the oil-coated ground layer sample (GB-03) by Py-GC/MS.

Peak No.	Retention Time (Min)	Compound	Relative Content (%)
1	5.62	Methyl pentanoate	2.48
2	8.15	Methyl hexanoate	0.59
3	10.85	Methyl heptanoate	0.78
4	13.44	Methyl octanoate	2.47
5	15.83	Methyl nonanoate	1.03
6	16.40	Dimethyl adipate	1.66
7	18.72	Dimethyl pimelate	1.25
8	20.97	Dimethyl suberate	5.52
9	23.23	Dimethyl azelate	31.03
10	24.92	Dimethyl sebacate	2.57
11	26.21	Methyl myristate	0.57
12	26.70	Dimethyl undecanedioate	1.09
13	27.97	Methyl pentadecanoate	0.27
14	29.81	Methyl palmitate	22.17
15	32.48	Methyl (Z)-9-octadecenoate	2.95
16	32.98	Methyl stearate	19.62
17	33.26	Alkylphenyl alkanoate (APAS), heat-bodied oil marker	1.89
18	36.10	Methyl dehydroabietate	0.42
19	38.43	Methyl behenate	0.61
20	39.31	Methyl 7-oxodehydroabietate	0.69
21	45.14	β-Sitosterol acetate	0.09
22	49.17	Stigmasta-3,5-dien-7-one	0.25

**Table 5:** Characteristic pyrolysis products detected in the polychrome painting sample (GB-13) by Py-GC/MS.

Peak No.	Retention Time (Min)	Compound	Relative Content (%)
1	3.89	1H-Pyrrole, 1-methyl-	0.61
2	9.24	Trimethyl phosphate	1.03
3	10.28	1,3,5-Triazine, hexahydro-1,3,5-trimethyl-	35.16
4	11.02	(1H)-Pyrrole-3-carbonitrile, 2-methyl-	0.33
5	11.29	Dimethyl succinate	8.77
6	11.42	Methyl 1-methyl-L-prolinate	0.66
7	13.39	Methyl 1-methylpyrrole-2-carboxylate	1.09
8	13.87	Dimethyl glutarate	4.03
9	16.40	Dimethyl adipate	2.08
10	17.72	Creatinine	3.31
11	17.92	1H-Indole, 3-methyl-2-(2-dimethylaminopropyl)-	3.11
12	18.41	Blood/egg white marker 5	0.19
13	18.68	Dimethyl pimelate	1.75
14	18.89	Methyl N-methoxycarbonyl-L-prolinate	4.44
15	20.57	N-Methoxycarbonyl-L-proline isohexyl ester	1.95
16	20.86	Dimethyl suberate	3.36
17	22.94	Dimethyl azelate	6.79
18	23.09	1-Methyl-3-hydrazono-1H-indole-2,3-dione	0.08
19	24.79	Dimethyl sebacate	1.55
20	26.14	Methyl myristate	0.82
21	26.54	Ethyl 1-ethylpyrrolidine-2-carboxylate	0.58
22	28.14	Methyl 5-hydroxy-1,2-dimethylindole-3-carboxylate	0.05
23	29.21	Methyl (Z)-9-hexadecenoate	0.94
24	29.69	Methyl palmitate	10.61
25	32.81	Methyl stearate	6.47
26	35.95	Methyl dehydroabietate	0.06
27	37.46	Methyl-7-methoxytetrahydroabietate	0.10
28	39.12	Methyl-7-oxodehydroabietate	0.05
29	39.42	Cholesta-2,4-diene	0.03

## Data Availability

The original contributions presented in this study are included in the article/Supplementary Materials. Further inquiries can be directed to the corresponding author.

## Supplementary Materials

The following supporting information can be downloaded at: https://www.mdpi.com/article/10.3390/ma19173672/s1, Figure S1: Blue pigment particles of GB-07 at 800× magnification; Figure S2: Scanning electron microscopy (secondary electron mode) images of GB-07 blue pigment particles at 10,000× magnification: (a) #1; (b) #2; (c) #3; (d) #4; Figure S3: Micrograph of blue particles at 50× magnification (plane-polarized light); Figure S4: EDS elemental analysis of GB-07 blue pigment particles.

## Abbreviations

The following abbreviations are used in this manuscript:

PLM	Polarized Light Microscopy
EDX	Energy-Dispersive X-ray Spectroscopy
SEM-EDS	Scanning Electron Microscopy with Energy-Dispersive Spectroscopy
Raman	Micro-Raman Spectroscopy
FT-IR	Fourier-Transform Infrared Spectroscopy
Py-GC/MS	Pyrolysis-Gas Chromatography/Mass Spectrometry
XRD	X-ray Diffraction
TIC	Total Ion Current Chromatogram
APAS	Alkylphenyl Alkanoate
