# Supplementary material for: Scientific Analysis of the Polychrome Paintings on the Stele Pavilion of Guandi Temple in Shanxi, China
Source: Materials (Basel). 2026 Aug 29;19(17):3672. doi: 10.3390/ma19173672 (PMC13566851; doi:10.3390/ma19173672)
Supplement: Supplementary file 1 [file materials-19-03672-s001.zip › materials-4487115-supplementary.pdf]

Supplementary Material

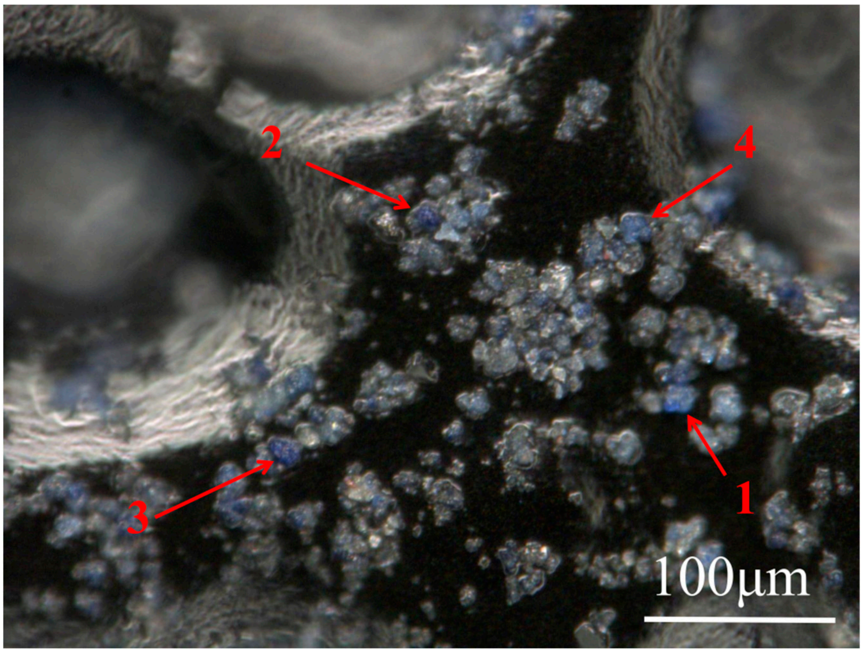

Figure S1. Blue pigment particles of GB-07 at 800× magnification.

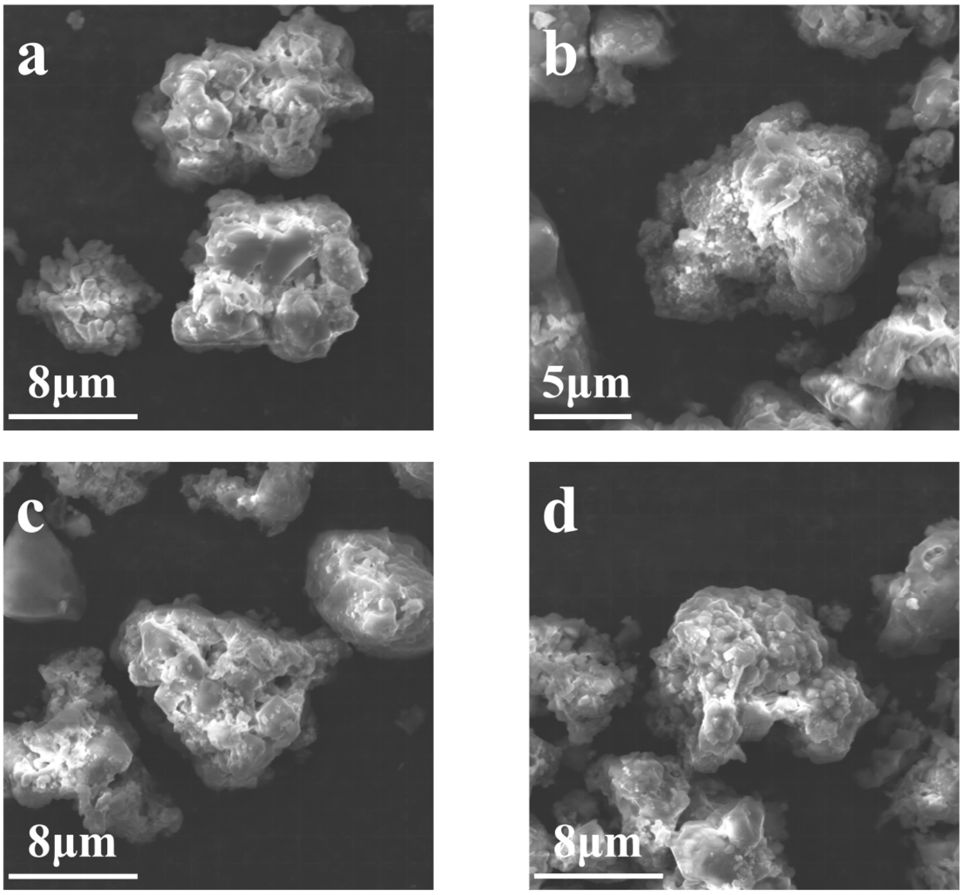

Figure S2. Scanning electron microscopy (secondary electron mode) images of GB-07 blue pigment particles at 10,000× magnification: (a) #1; (b) #2; (c) #3; (d) #4.

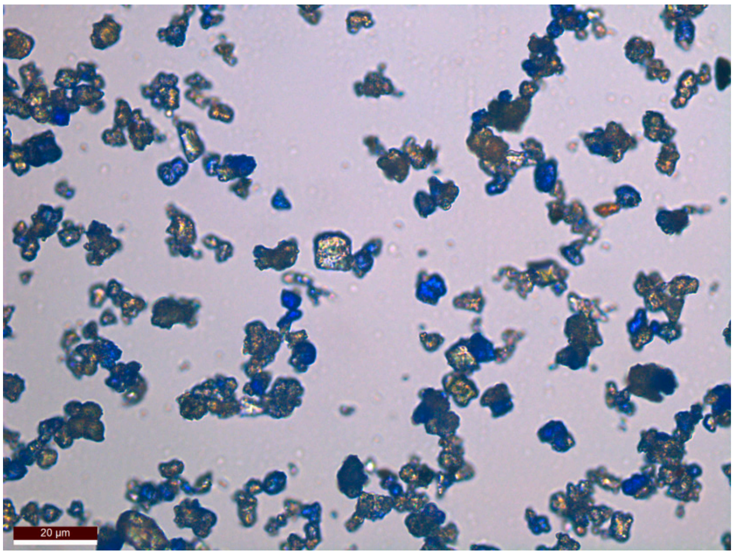

Figure S3. Micrograph of blue particles at 50× magnification (plane-polarized light).

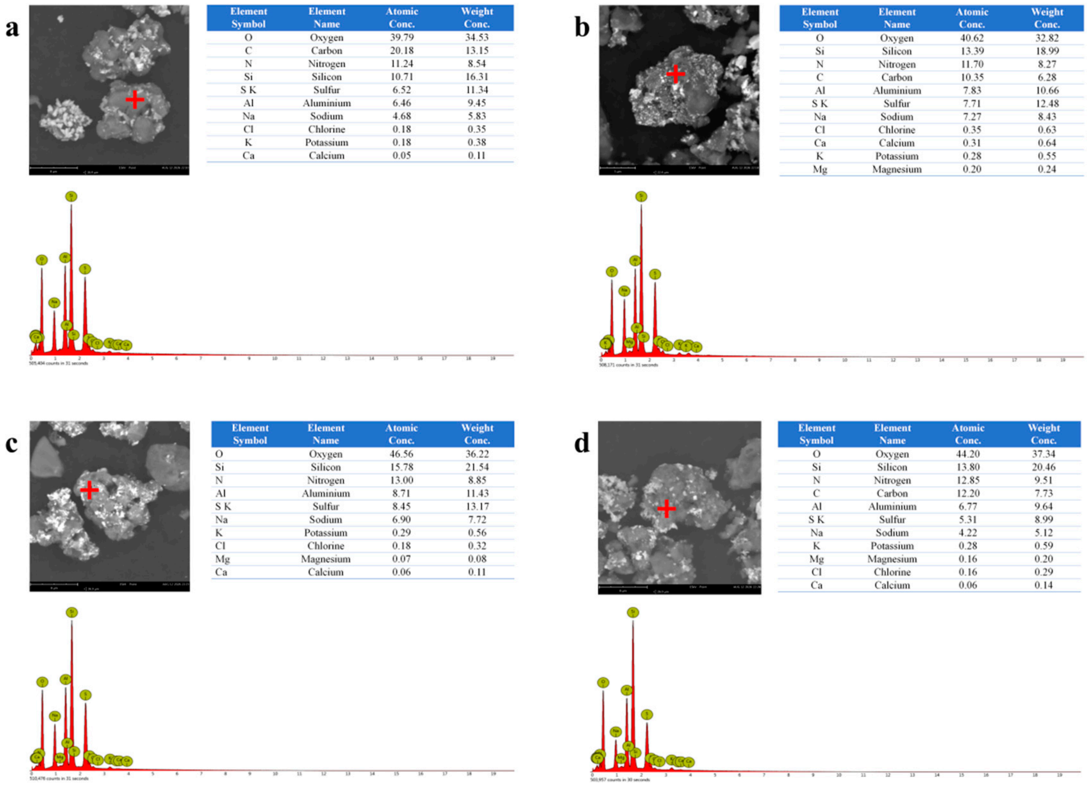

a

Element Symbol	Element Name	Atomic Conc.	Weight Conc.
O	Oxygen	39.79	34.53
C	Carbon	20.18	13.15
N	Nitrogen	11.24	8.54
Si	Silicon	10.71	16.31
S K	Sulfur	6.52	11.34
Al	Aluminium	6.46	9.45
Na	Sodium	4.68	5.83
Cl	Chlorine	0.18	0.35
K	Potassium	0.18	0.38
Ca	Calcium	0.05	0.11

b

Element Symbol	Element Name	Atomic Conc.	Weight Conc.
O	Oxygen	40.62	32.82
Si	Silicon	13.39	18.99
N	Nitrogen	11.70	8.27
C	Carbon	10.35	6.28
Al	Aluminium	7.83	10.66
S K	Sulfur	7.71	12.48
Na	Sodium	7.27	8.43
Cl	Chlorine	0.35	0.63
Ca	Calcium	0.31	0.64
K	Potassium	0.28	0.55
Mg	Magnesium	0.20	0.24

c

Element Symbol	Element Name	Atomic Conc.	Weight Conc.
O	Oxygen	46.56	36.22
Si	Silicon	15.78	21.54
N	Nitrogen	13.00	8.85
Al	Aluminium	8.71	11.43
S K	Sulfur	8.45	13.17
Na	Sodium	6.90	7.72
K	Potassium	0.29	0.56
Cl	Chlorine	0.18	0.32
Mg	Magnesium	0.07	0.08
Ca	Calcium	0.06	0.11

d

Element Symbol	Element Name	Atomic Conc.	Weight Conc.
O	Oxygen	44.20	37.34
Si	Silicon	13.80	20.46
N	Nitrogen	12.85	9.51
C	Carbon	12.20	7.73
Al	Aluminium	6.77	9.64
S K	Sulfur	5.31	8.99
Na	Sodium	4.22	5.12
K	Potassium	0.28	0.59
Mg	Magnesium	0.16	0.20
Cl	Chlorine	0.16	0.29
Ca	Calcium	0.06	0.14

Figure S4. EDS elemental analysis of GB-07 blue pigment particles.
